# Catastrophic Bone Cement Implantation Syndrome Treated with Venoarterial Extracorporeal Membrane Oxygenation: A Case Report

**DOI:** 10.1016/j.case.2021.10.003

**Published:** 2021-11-30

**Authors:** Valérian Valiton, Georgios Giannakopoulos, Hajo Müller

**Affiliations:** Department of Cardiology, Geneva University Hospital, Geneva, Switzerland

**Keywords:** Bone cement implantation syndrome, Cardiac arrest, Venoarterial extracorporal membrane oxygenation, Hip fracture, Case report

## Abstract

• BCIS is a rare but serious complication of cemented bone surgeries.
• The pathophysiology is suggested to be a pulmonary embolization of various particles.
• Cardiopulmonary conditions, femur fracture, and old age are risk factors of BCIS.
• Percutaneous VA ECMO may provide transient support for acute hemodynamic instability.

## Graphical abstract

**Figure:**
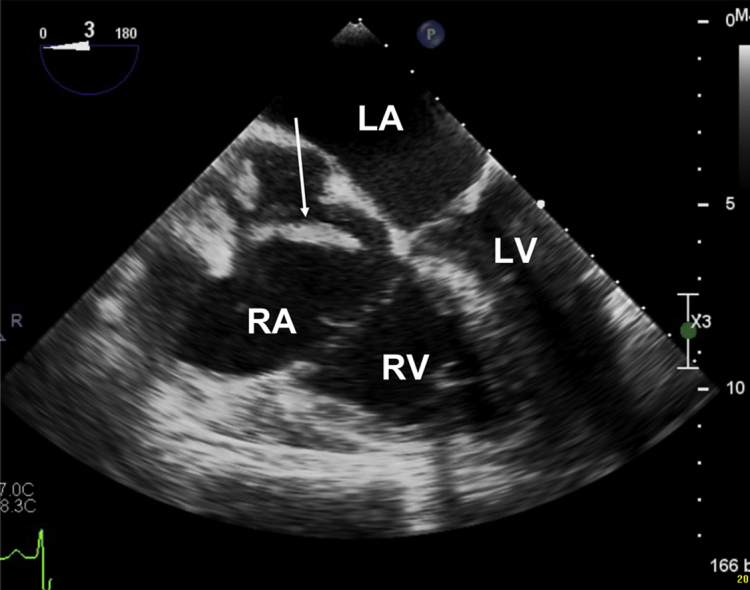

## Introduction

Bone cement implantation syndrome (BCIS) is a rare but potentially life-threating complication of orthopedic surgeries that use cement to hold a prothesis in place. It is characterized by sudden hypoxia, hypotension, or circulatory collapse occurring at the time of cementoplasty. The pathophysiology is poorly understood, but it is suspected that high intramedullary pressure during cementation leads to embolization of mixed contents (fat, air, marrow, aggregates of fibrin, or cement particles) in the pulmonary vasculature resulting in decreased cardiac output.^1^ The following is a case report of a 77-year-old woman with cardiac arrest due to BCIS treated with venoarterial extracorporeal membrane oxygenation (VA ECMO).

## Case Presentation

**Figure 1:**
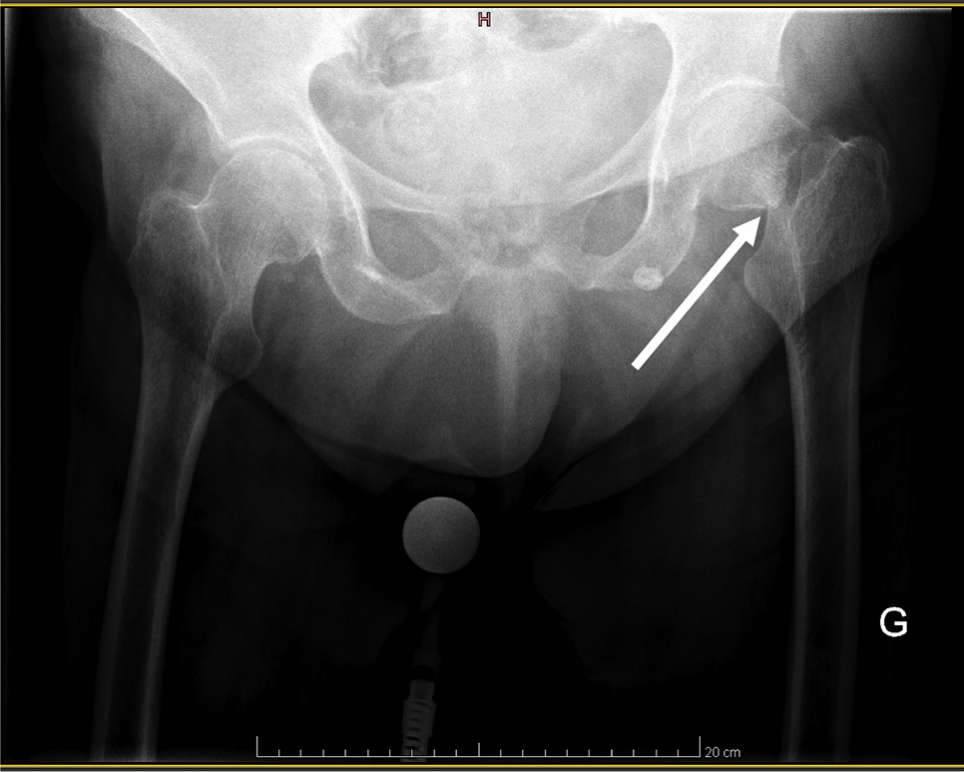
Plain radiography with anteroposterior pelvic projection showing a Garden IV left hip fracture with complete displacement (*white arrow*).

A 77-year-old woman was hospitalized following a fall on her left hip. Her medical history was significant for permanent atrial fibrillation, moderate pulmonary hypertension, and obstructive septal hypertrophic cardiomyopathy. She became symptomatic at the age of 58 years and had a maximal left ventricular outflow tract gradient of 90 mm Hg, so it was decided to perform a septal alcohol ablation. Because of an unsatisfactory result, a second ablation was conducted leading to complete regression of the intracardiac gradient. Her last transthoracic echocardiography performed 1 year earlier showed a normal left ventricular ejection fraction and absence of residual significant intracardiac gradient. The fall was provoked by intense fatigue and weakness following mild coronavirus disease (COVID-19). A Garden IV fracture (Figure 1) was diagnosed, and total hip replacement under spinal anesthesia was performed.

**Figure 2:**
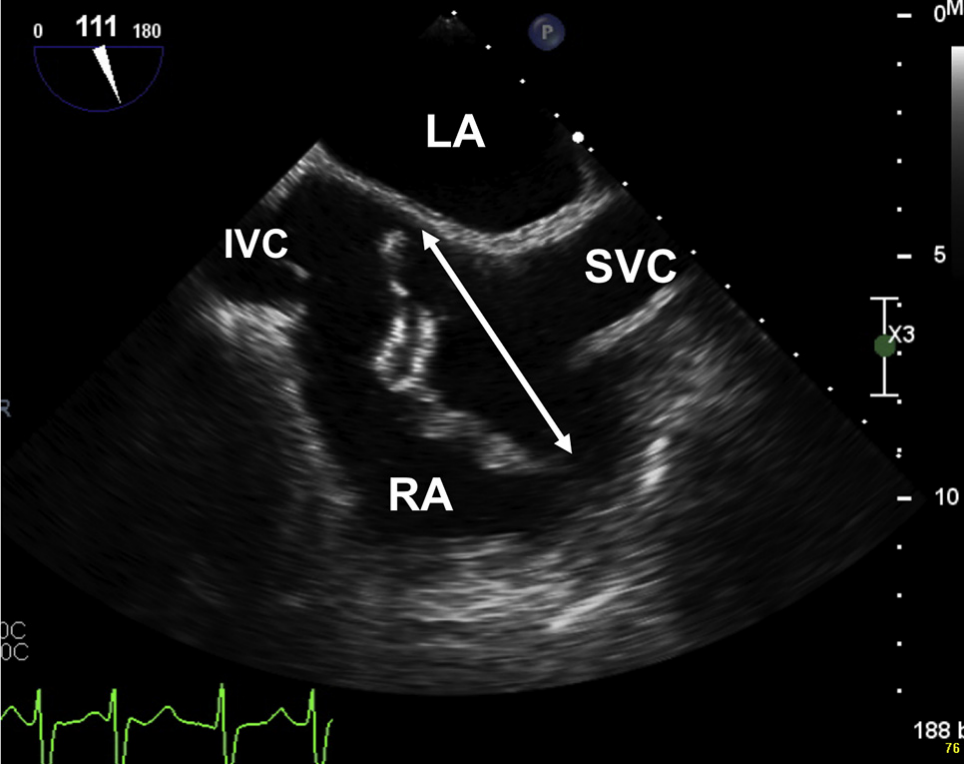
TEE midesophageal two-dimensional bicaval (111°) view immediately after cardiac resuscitation. Heterogeneous hyperechoic mobile content inside the right atrium is trapped within the Chiari network and measures 5.8 cm (*white double arrow*). *IVC*, Inferior vena cava; *LA*, left atrium; *RA*, right atrium; *SVC*, superior vena cava.

During the cementation of the prothesis, the patient went into sudden cardiac arrest. The initial rhythm was pulseless electrical activity. Cardiac resuscitation was promptly initiated, and the patient was intubated. Return of spontaneous circulation was achieved after 10 minutes of resuscitation, and the patient received a total of 5 mg of adrenaline. A rescue transesophageal echocardiogram (TEE) was rapidly performed and showed a large (4-5 cm) hyperechoic mobile mass in the right atrium (Figure 2, Video 1). This heterogeneous mass seemed to be attached to the Chiari network and expanded from the orifice of the inferior vena cava to the interatrial septum.

**Figure 3:**
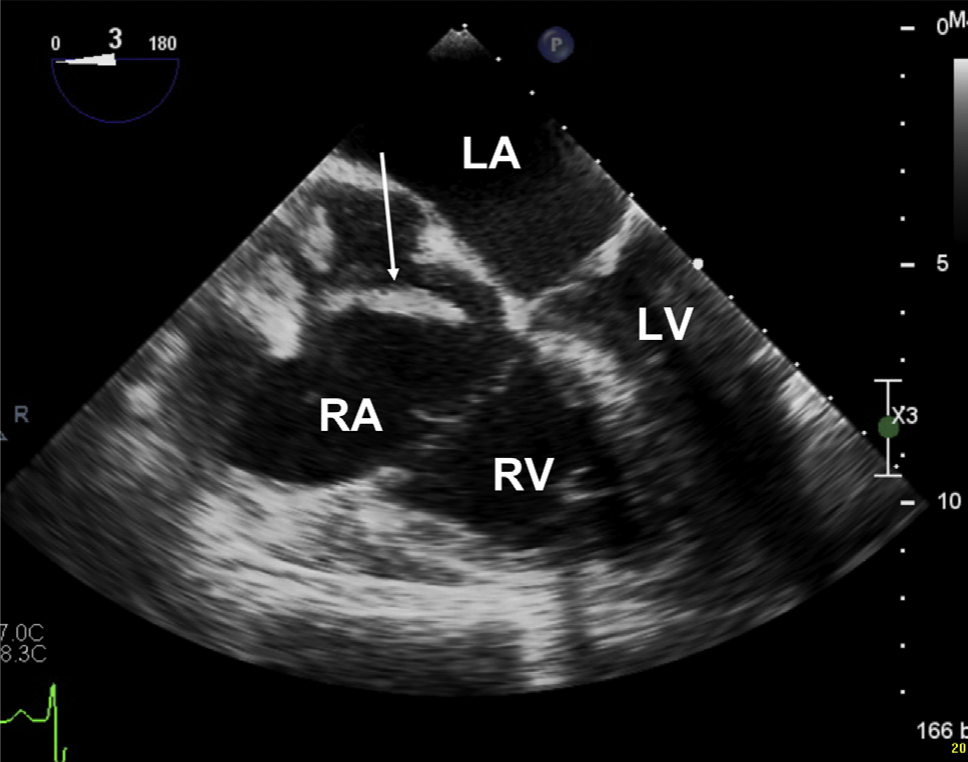
TEE midesophageal two-dimensional modified four-chamber (3°) view showing right atrial and ventricular dilatation after cardiac resuscitation. The hyperechoic mass is visible in the right atrium (*white arrow*). The interatrial septum is shifted toward left atrium due to increase right atrial pressure. *LA*, Left atrium; *LV*, left ventricle; *RA*, right atrium.

The left ventricle was hyperdynamic with a normal ejection fraction and without significant intraventricular gradient (peak gradient of 31 mm Hg); however, the right ventricle (RV) was dilated and had severely impaired function (Figure 3, Video 2). Moderate tricuspid regurgitation was also present.

**Figure 4:**
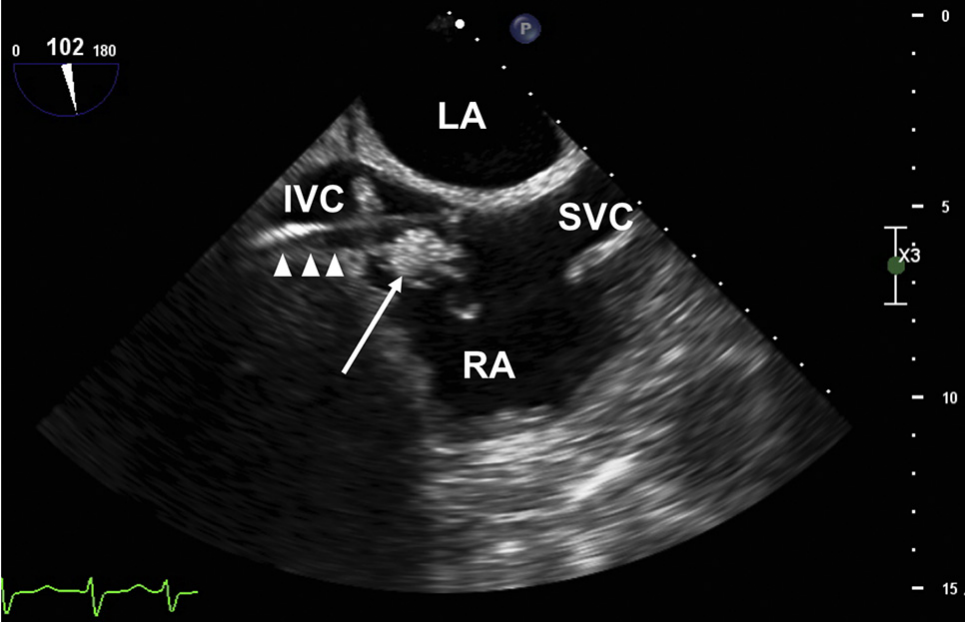
TEE midesophageal two-dimensional bicaval (102°) view after VA ECMO implantation. The hyperechoic mass (*arrow*) is trapped around the venous cannula of the VA ECMO (*arrowheads*). *LA*, Left atrium; *IVC*, inferior vena cava; *RA*, right atrium; *SVC*, superior vena cava.

**Figure 5:**
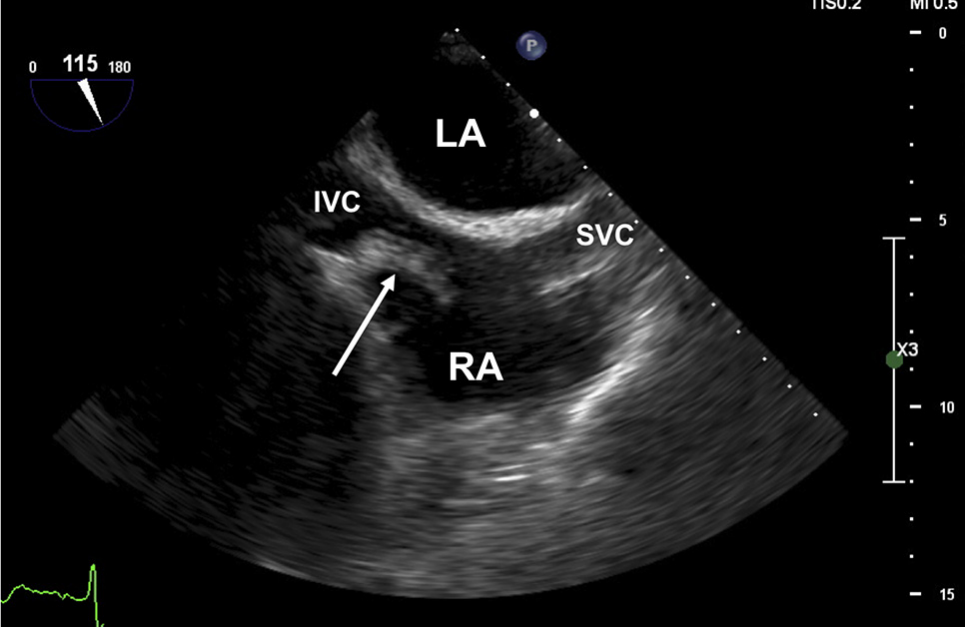
TEE midesophageal two-dimensional bicaval (115°) view showing regression of the hyperechoic mass (*white double arrow*) after 5 days of therapeutic anticoagulation and before VA ECMO withdrawal. The mass is still trapped within the Chiari network albeit reduced in size and measuring 2.8 cm. *LA*, Left atrium; *IVC*, inferior vena cava; *RA*, right atrium; *SVC*, superior vena cava.

High doses of continuous intravenous epinephrine were initiated (0.5 γ/kg/minute), but the patient remained hemodynamically unstable. After multidisciplinary discussion, it was decided to implant a VA ECMO. Following the procedure, the echoic mass was trapped around the venous canula inside the right atrium (Figure 4, Video 3). Seven days later, repeat TEE showed regression of the mass size under therapeutic anticoagulation (Figure 5). The right ventricular (RV) function improved with a near-to-normal radial contractility (Video 4). The patient was successfully weaned from VA ECMO. Despite aspiration from the venous canula during VA ECMO removal, the right atrial mass was still present, measuring 2.8 cm with a long filament on it (Video 5).

The patient remained hemodynamically stable in the following days; however, an unfavorable neurological outcome was observed. Four days after VA ECMO withdrawal, the patient developed progressive respiratory failure, fever, and elevated inflammatory biomarkers. A computed tomography demonstrated a pattern most consistent with infection but without pulmonary embolism. Pulmonary septic shock was suspected, and broad-spectrum antibiotics were initiated. The patient did not respond well to the initial therapy and started to become hypotensive with signs of circulatory failure and eventually multiple organ dysfunction syndrome, so it was decided to withdraw care. Since the mass remained after VA ECMO withdrawal, a recurrent pulmonic embolic event cannot be entirely excluded as a contributing factor even if the lung computed tomography did not show any pulmonary embolism.

## Discussion

The exact prevalence of BCIS remains unknown as it is a rare condition. The largest retrospective study includes 22,000 patients in the United Kingdom with hemiarthroplasty following neck of femur fractures.^2^ It has been reported that BCIS occurs in one out of every 2,900 (0.03%) hemiarthroplasties, with a high mortality rate of up to 66% of patients. However, these figures may underestimate the true incidence, as only severe cases were reported. It is suggested that neck of femur fractures, preexisting cardiopulmonary dysfunction, old age, and pulmonary hypertension are all risk factors for developing BCIS. All of these risk factors were present in the patient in this case report. Specific anesthetic and surgical measures should be taken to reduce the risk in patients presenting with the aforementioned risk factors.^1^

We want to highlight the importance and value of rescue TEE in our patient as it led to prompt diagnosis of the hemodynamic instability. Rescue TEE is defined by unplanned TEE examination performed in an emergent setting to diagnose causes of hemodynamic instability or cardiopulmonary arrest in the perioperative setting.^3^ Rescue TEE is useful in many situations where rapid diagnosis is needed for refractory hypotension, arrhythmias, evidence of myocardial ischemia, severe hypoxemia, or cardiac arrest, as depicted in our case. In a systematic review of 321 studies with 400 patients with noncardiac intraoperative hemodynamic compromise, the most frequent diagnoses of rescue TEE or transthoracic echocardiography were hypovolemia (33.2%), low ejection fraction (20.5%), RV failure (13.1%), acute coronary syndrome (10.1%), and pulmonary embolism (5.8%).^4^

There is no widely approved definition and classification of BCIS. Donaldson *et al*^1^ suggest the following definition: “BCIS is characterized by hypoxia, hypotension or both and/or unexpected loss of consciousness occurring around the time of cementation, prosthesis insertion, reduction of the joint or, occasionally, limb tourniquet deflation in a patient undergoing cemented bone surgery.” A classification is proposed and ranges from grade I with moderate hypoxia and hypotension (SaO2 < 94% and fall in systolic blood pressure > 20%) to grade III with cardiac arrest.^1^

Various hypotheses have been proposed regarding the pathophysiology of BCIS. The most widely accepted is the “embolization model,” which suggests that an increase in intramedullary pressure at cementation leads to the embolization of either fat, fibrin, platelets, cement, air, or bone particles.^1^ Our echocardiography findings, which displayed hyperechoic content, are highly suggestive of this proposed mechanism.

The embolization induces obstruction of the pulmonary circulation with an increase in the pulmonary vascular resistance. This leads to RV ejection fraction impairment and reduced cardiac output.^5^

## Conclusion

BCIS is a rare but serious and potentially fatal complication of cemented total hip replacement and hemiarthroplasties. Its occurrence is difficult to predict, but preexisting cardiopulmonary conditions, neck of femur fractures, and old age are considered risk factors. This case suggests that grade III BCIS during hip hemiarthroplasty can be temporarily stabilized with percutaneous VA ECMO.

## Supplementary Data

Video 1 TEE midesophageal two-dimensional bicaval (104°) view performed immediately after cardiac resuscitation showing a hyperechoic heterogeneous mobile mass in the right atrium. The mass seems to be trapped within the Chiari network and attached to the inferior vena cava. The size of this highly mobile mass is between 5 and 6 cm.

Video 2 TEE midesophageal two-dimensional modified four-chamber (2°) view showing right atrial and ventricular dilatation after cardiac resuscitation. The RV function is severely impaired. The hyperechoic mobile mass is visible in the right atrium. The interatrial septum is shifted toward the left atrium due to increase right atrial pressure.

Video 3 TEE midesophageal two-dimensional bicaval (102°) view after VA ECMO implantation. The hyperechoic mass is trapped around the venous cannula of the VA ECMO. Despite aspiration by the venous cannula during the intervention, the mass was still present after implantation.

Video 4 TEE midesophageal two-dimensional four-chamber (0°) view 4 days after cardiac arrest showing near-to-normal RV radial contractility. This view is different from Video 2; therefore, the comparison of RV function should be taken with caution. The mass is less mobile and visible in the right atrium, measuring 2.8 cm with a long filament of 3 cm on it.

Video 5 TEE midesophageal two-dimensional bicaval (115°) view during withdrawal of VA ECMO venous cannula. The venous cannula is mobilized in the right atrium, but despite aspiration, the hyperechoic mass is still present.

## References

[1] Donaldson A.J., Thomson H.E., Harper N.J., Kenny N.W. (2009). Bone cement implantation syndrome. Br J Anaesth.

[2] Rutter P.D., Panesar S.S., Darzi A., Donaldson L.J. (2014). What is the risk of death or severe harm due to bone cement implantation syndrome among patients undergoing hip hemiarthroplasty for fractured neck of femur? A patient safety surveillance study. BMJ Open.

[3] Hofer C.K., Mizuguchi A.K., Popescu W.M. (2012). Monitoring the patient at risk of hemodynamic instability in remote locations. Int Anesthesiol Clin.

[4] Jasudavisius A., Arellano R., Martin J., McConnell B., Bainbridge D. (2016). A systematic review of transthoracic and transesophageal echocardiography in non-cardiac surgery: implications for point-of-care ultrasound education in the operating room. Can J Anaesth.

[5] Ereth M.H., Weber J.G., Abel M.D., Lennon R.L., Lewallen D.G., Ilstrup D.M. (1992). Cemented versus noncemented total hip arthroplasty—embolism, hemodynamics, and intrapulmonary shunting. Mayo Clin Proc.

